# Preeclampsia in a pregnant woman with severe aplastic anemia: A case report

**DOI:** 10.1002/ccr3.6789

**Published:** 2022-12-26

**Authors:** Ikuno Kawabata, Ryoko Yokote, Sayuri Kasano, Jun Ogawa, Masahiko Kato, Tomoko Ichikawa, Mirei Yonezawa, Yoshimitsu Kuwabara, Hiroki Yamaguchi, Shunji Suzuki

**Affiliations:** ^1^ Department of Obstetrics and Gynecology Nippon Medical School Tokyo Japan; ^2^ Department of Hematology Nippon Medical School Tokyo Japan

**Keywords:** eltrombopag, fetal growth restriction, preeclampsia, severe aplastic anemia

## Abstract

A pregnant woman with severe aplastic anemia was managed using biweekly red blood cell transfusion and oral eltrombopag olamine administration during pregnancy. She was diagnosed with preeclampsia at 35 weeks of gestation. The severity of aplastic anemia is very important for predicting the course of pregnancy.

## Abstract 2

The frequency of preeclampsia in pregnant women with aplastic anemia may be higher than that in general pregnancies. Attention is paid to severe anemia in the early trimesters. Eltrombopag can be used in pregnancy.

## 1 INTRODUCTION

Aplastic anemia (AA) is a type of bone marrow failure syndrome defined by the presence of pancytopenia in the absence of abnormal infiltrates or bone marrow fibrosis.^1^ Pregnant women with AA experience multiple and serious risks for both the mother and fetus, such as postpartum hemorrhage, puerperal sepsis, acute heart failure, severe preeclampsia, gestational diabetes mellitus, miscarriage, preterm birth, preterm premature rupture of membrane (pPROM), stillbirths, and fetal growth restriction.^2^, ^3^

Preeclampsia is a complication of pregnancy characterized by high blood pressure and signs of damage to other organ systems after 20 weeks of gestation. Thrombocytopenia is also associated with preeclampsia. When women with AA develop preeclampsia during pregnancy, their condition may be serious. However, there are limited data on the rare co‐occurrence of AA and preeclampsia, and the exact relationship between AA and preeclampsia remains unclear.

In this case report, we describe a pregnant woman with severe AA who developed preeclampsia.

## 2 CASE

A 35‐year‐old primiparous woman presented to our hospital at 6 weeks of gestation. At 23 years, she had been diagnosed with acquired AA that was refractory to immunosuppressive therapy, including cyclosporine and corticosteroid therapy. Her disease was without a known cause, not secondary as drug‐induced and radiation. Her bone marrow findings 7 years ago were hypoplastic and no dysplastic cells and cellularity was about 20%. She achieved spontaneous pregnancy, and at the initial visit, her blood pressure was normal and her proportion was normal instead of obesity. However, she was stable even without treatment, with pre‐pregnancy hemoglobin (Hb) levels and platelet count of 9–10 g/dl and 20,000–25,000/ml, respectively. At 8 weeks of gestation, her Hb level, white blood cell count, and platelet count had decreased 6.9 g/dl, 2900/ml, and 15,000/ml, respectively. During her next visit at 9 weeks of gestation, her Hb level and platelet count were 6.6 g/dl and 14,000/ml, respectively.

Therefore, after 9 weeks of gestation, she underwent red blood cell transfusion every 1–2 weeks to maintain her Hb level >8.0 g/dl and was started on oral eltrombopag olamine (25 mg once daily) for severe thrombocytopenia. Her Hb level and platelet count were maintained at 8–10 g/dl and 14,000–18,000/ml, respectively, up to 32 weeks of gestation. We could not detect any fetal abnormality on ultrasound screening during the second trimester, except that the umbilical cord was inserted in the fetal membranes. Weekly and fortnightly ultrasonography revealed that the estimated fetal weight was within the normal range for gestational age. There were no abnormal changes in the fetal biophysical profile, amniotic fluid volume, or umbilical artery Doppler velocimetry until 30 weeks of gestation. Her blood pressure was slightly elevated at 130–140 /80–90 mmHg above after 30 weeks of gestation. Fetal growth restriction was observed by ultrasound at 32 weeks of gestation, and the growth was arrested during the next 2 weeks. Her Hb increased to 11 g/dl without red blood cell transfusion, while the platelet counts slightly decreased from 11,000 to 12,000/ml. At 34 weeks and 5 days, fetal weight was estimated to be 1448 g, with a high umbilical artery pulsatility index (>95th percentile). Her blood pressure increased to 150/80 mmHg for the first time; the urine protein/creatine ratio was 1.26 g/g Cr, while the platelet count decreased to 9000/μl. Her liver enzymes were within normal range, and serum creatinine was 0.68 mg/dl. She was admitted for close monitoring. After admission, her blood pressure rose to above 160/100. Transient cardiotocography showed severe variable decelerations, occasionally in the absence of uterine contractions. With these major findings (maternal hypertension, urine protein, and uteroplacental dysfunction) a diagnosis of preeclampsia was made. Transient cardiotocography showed severe variable decelerations, occasionally in the absence of uterine contractions. With these major findings, a diagnosis of severe preeclampsia was made and decided to terminate the pregnancy.

After platelet transfusion, she delivered a 1348 g female baby via cesarean section at 35 weeks and 0 day under general anesthesia. Intraoperative hemorrhage was 670 ml; she had no intraoperative or postpartum complications. Her baby did not develop respiratory distress syndrome and had mild transient tachypnea of newborn. The baby needed only mild respiratory support with a nasal cannula in room air. Neonatal laboratory data did not show pancytopenia. The baby was discharged 1 month after birth, and the follow‐up was normal at 18 months. The mother was treated with nicardipine injection from the preoperative period to the first day postpartum, followed by oral nifedipine administration for about 2 weeks to strictly control the blood pressure. Within 1 month postpartum, her blood pressure normalized without medication, and urine protein became undetectable. Her Hb level and platelet count had returned to the pre‐pregnancy level at 2 weeks postpartum.

## 3 DISCUSSION

In this case, a woman with severe AA developed preeclampsia. Her platelet count was <20,000/ml at the onset of pregnancy and significantly decreased to critical level with the onset of preeclampsia at 34 weeks of gestation.

In AA, stem cells in the bone marrow are damaged by various causes, and the bone marrow is either empty or contains few blood cells and cannot produce blood cells. Therefore, in pregnant women with AA, physicians must watch for infection due to fewer white blood cells, severe anemia due to fewer red blood cells, and intra‐postpartum bleeding due to fewer platelets in addition to common pregnancy complications. A previous retrospective study in pregnant women with AA2 demonstrated that 16.7% of pregnancies were uneventful, but 83.3% had complications such as premature labor (33.3%), gestational diabetes (18.3%), preeclampsia (16.7%), acute heart failure (5.0%), pPROM (3.3%), pregnancy and postpartum hemorrhage (5.0%), and postpartum infection (1.7%). Chen et al.^3^ demonstrated that the rate of complications in pregnant women with AA was 53.3%. Shin et al.^4^ reported that severe thrombocytopenia (<20,000/μl) is more associated with obstetric and disease complications than non‐severe thrombocytopenia in pregnant women with AA. Therefore, pregnancy‐complicated AA poses an increased risk for both the mother and fetus.

When pregnant women with AA develop severe preeclampsia, platelet counts decrease to a potentially critical level. Few studies have demonstrated the relationship between acquired bone marrow failure syndromes, such as AA and myelodysplastic syndrome (MDS), and preeclampsia. Bo et al.^2^ showed, in a retrospective analysis of 60 pregnant women with AA, that 10 women (16.7%) developed preeclampsia. In a retrospective study, Yang et al.^5^ reported that among 25 pregnant women with MDS, six (24%) developed hypertensive disorders of pregnancy, five of which were cases of severe preeclampsia. Preeclampsia complicates 2%–8% of all pregnancies globally^6^; therefore, the frequency of preeclampsia in pregnant women with acquired bone marrow failure may be higher than that in the general population. Pregnancy with aplastic anemia is rare and some reports published, and there is a similar case report as our case. Wong et al.^7^ reported a case report who developed preeclampsia at 25 weeks of gestation. hemoglobin of the case was 6.4 g/dl. Some reports have recently suggested an association between severe anemia and preeclampsia however the reason for this is unclear.

Chen et al.^3^ demonstrated that severe anemia (Hb <7.0 g/dl) during pregnancy is significantly associated with preeclampsia and eclampsia (adjusted odds ratio [aOR] 3.74, 95% confidence interval [CI]: 2.90–4.81 in nulliparous and aOR 3.45, 95% CI 2.79–4.25 in multiparous women). Smith et al.^8^ showed that the incidence of preeclampsia was high among pregnant women with anemia. Because severe anemia is a characteristic of bone marrow failure, it may have contributed to the occurrence of preeclampsia. Although the relationship between severe anemia in pregnancy and preeclampsia remains unclear, it is thought that the decreased oxygen‐carrying capacity of blood leads to hypoxemia and hypoxia in tissues, which may be a common factor inducing angiogenesis. Recent studies have shown that hypoxia stimulates the secretion of antiangiogenic factors, which play a harmful role in spiral artery remodeling, leading to the development of preeclampsia.^9^, ^10^ Stangret et al.^11^ demonstrated increased expression of Flt‐1 under mild anemia and morphological changes in the placenta of women with mild anemia, such as increased fetal villous capillarization, greater volume and diameter of the villus, and an increased number of capillaries per villus cross‐section.^12^ However, the effects of angiogenic factor secretion may differ between mild and severe anemia because of the different degrees of hypoxia in tissues. It is unclear how maternal severe anemia in the presence of hypoxic conditions affects the secretion of antiangiogenic factors for placental formation. In this case, the patient was transfused with red blood cells every 1–2 weeks, and her Hb was >9–10 g/dl after 10 weeks of gestation. However, the anemia worsened at 6–9 weeks of gestation—consistent with the implantation time frame. The increase in plasma volume begins at approximately 6 weeks of gestation and naturally causes physiological hemodilution. Most pregnant women in early trimesters do not develop anemia because of resilience in their bone marrow; however, women with AA may not recover due to bone marrow insufficiency. Therefore, we recommend that attention be paid to anemia occurring in pregnant women with AA in the early trimesters, as the early treatment of anemia may prevent the development of preeclampsia.

Eltrombopag, a thrombopoietin‐receptor agonist (TPO‐Ra), has proven to be effective in managing immune thrombocytopenic purpura in clinical studies, but its safety in pregnancy remains uncertain.^13^ In our case, the platelet count was <20,000/ml at the onset of pregnancy. Platelet counts in most women begin to decrease in the mid‐second to the third trimester and continue to decrease until delivery because of increased platelet turnover and plasma dilution, immune‐mediated mechanisms, or a complication such as preeclampsia. Supportive care is recommended to maintain Hb >8.0 g/dl and platelet count at 20,000/ml in pregnant women with AA.^13^, ^14^ We diagnosed no bleeding tendency symptoms in this case. Therefore, we treated our patient with TPO‐Ras to prevent further decrease in the platelet count. Howaidi et al.^13^ suggested an association between eltrombopag administration in the first to the second trimester and low fetal birth weight. In our case, it is unclear whether eltrombopag had any adverse effects, such as causing a reduction in birth weight, since the influence of umbilical cord insertion and the development of preeclampsia are likely to be significant.

## 4 CONCLUSION

Here, we reported the case of a pregnant woman with severe AA who developed preeclampsia. Fewer reports of complications of both aplastic anemia and preeclampsia, but the frequency of preeclampsia in pregnant women with acquired bone marrow failure reported higher than that in general pregnancies. Severe anemia in early first trimester may potentially related to develop preeclampsia. We recommend that anemia occurring in early trimesters be carefully monitored and managed in pregnant women with AA. In our case, eltrombopag did not have harmful effects for pregnant women, but her baby's birth weight was small for gestational age. It is unclear whether eltrombopag had a causing a reduction in birth weight.

## AUTHOR CONTRIBUTIONS

IK contributed to the clinical management of the patient, and drafted the manuscript and contributed substantially to its revision. RK, SY, JO, MK, TI, and MY contributed to the clinical management of the patients. YK, HY, and SS were involved supervision and critical revision of this manuscript. All authors contributed to the preparation of this case report and have read and approved the final manuscript.

## FUNDING INFORMATION

No funding from an external source supported the publication of this case report.

## CONFLICT OF INTEREST

The authors declare that they have no conflict of interest regarding the publication of this case report.

## ETHICAL APPROVAL

This study has been approved by the ethics committee of Nippon Medical School Hospital (B‐2021‐465).

## CONSENT

The patient provided written informed consent for this publication.

## Data Availability

The data that support the findings of this study are available from the corresponding author upon reasonable request.
